# Supplementary material for: Colorectal Neoplasia Detection Rates in Lynch Syndrome
Source: Cancers (Basel). 2024 Nov 30;16(23):4021. doi: 10.3390/cancers16234021 (PMC11640722; doi:10.3390/cancers16234021)
Supplement: Supplementary file 1 [file cancers-16-04021-s001.zip › cancers-3322875-supplementary.pdf]

Supplemental Table S1. Colorectal neoplasia detection rates (%) stratified by age.

	Overall	<30	30-39	40-49	50-59	≥60
**CNDR**	27.9	28.1	13.9	22.2	28.2	41.8
**ADR**	21.4	19.3	8.4	16.2	21.8	34.9
Non-advanced	17.9	14.0	7.4	14.0	18.0	29.6
Advanced	5.4	6.1	1.0	3.1	5.4	10.2
**PSDR**	7.7	7.9	5.9	8.3	5.4	10.2
Non-advanced	5.1	4.4	5.0	5.1	3.4	6.9
Hyperplastic polyp	2.4	3.5	0.5	2.8	1.7	3.3
Advanced	0.7	0.0	1.0	0.9	0.7	0.6
**CRCDR**	1.5	1.8	0.5	0.9	2.4	2.1

CNDR: Colorectal Neoplasia Detection Rate
ADR: Adenoma Detection Rate
PSDR: Proximal Serrated Detection Rate
CRCDR: Colorectal Cancer Detection Rate

Supplemental Table S2. Risk factors associated with colorectal serrated lesion detection.

	Serrated lesion present (n = 77)	Serrated lesion absent (n = 277)	p
Biological sex (%)			0.62
Female	52 (67.5)	175 (63.6)	
Male	25 (32.5)	100 (36.4)	
Median age (%)			0.90
<30	8 (10.4)	30 (10.9)	
30-39	13 (16.9)	60 (21.8)	
40-49	18 (23.4)	58 (21.1)	
50-59	15 (19.5)	53 (19.3)	
≥60	23 (29.9)	74 (26.9)	
Race (%)			0.64
White	70 (90.9)	238 (86.5)	
Black	1 (1.3)	11 (4.0)	
Other	4 (5.2)	19 (6.9)	
Not reported	2 (2.6)	7 (2.5)	
Marital Status (%)			0.18
Single	14 (18.2)	63 (22.9)	
Married	51 (66.2)	188 (68.4)	
Divorced/Widowed/Other	12 (15.6)	24 (8.7)	
Insurance Type (%)			0.78
Private	59 (76.6)	209 (76.0)	
Medicare	16 (20.8)	53 (19.3)	
Medicaid	2 (2.6)	10 (3.6)	
Other	0 (0.0)	3 (1.1)	
Income (%)			0.91
0-49,999	3 (3.9)	14 (5.1)	
50,000-74,999	7 (9.1)	36 (13.1)	
75,000-99,999	17 (22.1)	62 (22.5)	
100,000-124,999	30 (39.0)	91 (33.1)	
125,000-149,999	12 (15.6)	38 (13.8)	
≥150,000	8 (10.4)	33 (12.0)	
Smoking status (%)			0.63
Never	49 (63.6)	191 (69.5)	
Former	24 (31.2)	72 (26.2)	
Current	4 (5.2)	12 (4.4)	
BMI at first colonoscopy/sigmoidoscopy (median [IQR])	26.6 [23.5, 31.5]	26.5 [23.1, 30.5]	0.65
ASA use ≥ 2 years (%)	31 (40.3)	114 (41.5)	0.95
Gene (%)			0.90
MLH1	19 (24.7)	57 (20.7)	
MSH2/EPCAM	24 (31.2)	88 (32.0)	
MSH6	15 (19.5)	59 (21.5)	

PMS2	19 (24.7)	71 (25.8)	
Surveillance interval in years (median [IQR])	1.2 [1.0, 1.5]	1.1 [1.0, 1.9]	0.43
History of prior colon resection (%)	20 (26.0)	73 (26.5)	1
Personal history of any prior cancer (%)	45 (58.4)	143 (52.0)	0.38
Personal history of prior colon cancer (%)	16 (20.8)	61 (22.2)	0.91
Personal history of other cancer (%)	37 (48.1)	109 (39.6)	0.23
Family history of any cancer (%)	75 (97.4)	267 (97.1)	1.00
Family history of colon cancer (%)	56 (72.7)	206 (74.9)	0.81
Family history of other cancer (%)	73 (94.8)	244 (88.7)	0.17
Number of colonoscopies/sigmoidoscopies			0.09
1	12 (15.6)	92 (33.5)	
2	12 (15.6)	52 (18.9)	
3	12 (15.6)	30 (10.9)	
4	12 (15.6)	30 (10.9)	
5	9 (11.7)	19 (6.9)	
6	8 (10.4)	13 (4.7)	
≥7	12 (15.6)	39 (14.3)	
